# Association between lactate dehydrogenase to albumin ratio and ICU mortality in patients with acute kidney injury: a retrospective cohort study

**DOI:** 10.3389/fneph.2025.1583913

**Published:** 2025-06-02

**Authors:** Jianting Gao, Huizhen Chen, Yiyi Wu, Chang Xu, Yan Jin

**Affiliations:** Intensive Care Unit, Hangzhou Hospital of Traditional Chinese Medicine, Hangzhou, China

**Keywords:** lactate dehydrogenase, albumin, lactate dehydrogenase to albumin ratio, acute kidney injury, mortality

## Abstract

**Background:**

Acute kidney injury (AKI) is a prevalent and severe medical condition that is frequently observed in the intensive care unit (ICU). Although numerous biomarkers have been identified to predict the prognosis of AKI, the lactate dehydrogenase to albumin ratio [LDH/ALB ratio (LAR)] has not been extensively investigated. The principal objective of this study was to assess the relationship between LAR and all-cause mortality in patients with AKI.

**Methods:**

A total of 6,831 AKI patients were included in this study, divided into survival (n = 5,152) and non-survival groups (n = 1,679). The association between LAR and mortality was examined through restricted cubic spline (RCS) analysis and Cox regression analysis. Subgroup analysis was used to search for interactive factors. Additionally, the prognostic capability of LAR was further evaluated using receiver operating characteristic (ROC) curve analysis.

**Results:**

The LAR was remarkably higher in the non-survival group (*p* < 0.001). RCS indicated a non-linear correlation between LAR and ICU death (*p* for non-linearity < 0.001). A LAR of 10.4 was used as the cutoff point to generate the high-LAR and low-LAR subgroups, and the Kaplan–Meier curves revealed that the ICU cumulative survival rate for patients with AKI was significantly lower in the high-LAR group (log-rank p < 0.001). The LAR’s prediction of ICU mortality in AKI patients yielded an area under the ROC curve of 0.65.

**Conclusion:**

Our research suggests that LAR monitoring may be promising as a prognostic marker among patients with AKI. Higher LAR is associated with greater ICU mortality.

## 1 Introduction

Acute kidney injury (AKI) is a commonly encountered clinical syndrome that is common in hospitalized patients, particularly those who are critically ill with sepsis or who have undergone major surgery. The “Epidemiology of acute kidney injury in critically ill patients” (AKI-EPI) study was an international cross-sectional study that used the complete Kidney Disease: Improving Global Outcomes (KDIGO) criteria to diagnose and stage AKI and showed that AKI occurred in more than 50% of intensive care unit (ICU) patients (1). In addition, the AKI-EPI study as well as others demonstrated that the development of AKI is associated with increased short- and long-term morbidity and mortality (2).

Clinicians may promote early intervention to minimize mortality using clinical signs to predict illness severity and prognosis. Thus, identifying high-risk AKI individuals is essential for timely and effective interventions to improve patient outcomes. In recent years, AKI has received much attention, and acute biomarkers have been investigated in AKI prediction and treatment (3–5), but applying these biomarkers in clinical practice still faces significant limitations (6).

Ischemia–reperfusion injury (I/R-I) is a leading cause of AKI in several disease states; renal tubular epithelial cells (RTECs) first produce energy via glycolysis rather than oxidative phosphorylation, resulting in a substantial conversion of pyruvate to lactate (7). Lactate dehydrogenase (LDH), as a common enzyme involved in energy metabolism in cells, is involved in this process (8, 9). Albumin (ALB), produced by the liver, is essential in various physiological processes, including antioxidation, anti-inflammation, and the regulation of plasma osmolality (10). Podocyte injury can disrupt the integrity of the glomerular filtration barrier, leading to proteinuria and hypoproteinemia (11). Serum albumin has been demonstrated to be correlated with the development of AKI and death after AKI (12, 13).

The LDH/ALB ratio (LAR) is emerging as a novel biomarker for critically ill individuals. While previous research on LAR has been centered around malignant tumors (14, 15), recent evidence suggests its relevance to the prognosis of patients with sepsis, sepsis-associated acute kidney injury (SA-AKI), pneumonia, and acute respiratory distress syndrome (ARDS) (16–19). The current research exploring its association with AKI patient outcomes in the ICU is not extensive. Consequently, this study was designed to examine the relationship between LAR and ICU mortality, with the objective of assessing the predictive value of LAR and providing guidance for clinical management.

## 2 Materials and methods

### 2.1 Study population

This was a retrospective observational cohort study that was conducted utilizing the Medical Information Mart for Intensive Care IV (MIMIC-IV, version 3.0) database. MIMIC-IV 3.0 is a comprehensive and freely accessible repository of intensive care data that encompasses over 94,000 ICU admissions at the Beth Israel Deaconess Medical Center in Boston, Massachusetts, from 2008 to 2022.

We obtained authorization to access the database (certification number: 55670126). All protected health information in the MIMIC database has been de-identified, so individual patient consent was not needed.

The definition of AKI was according to the criteria of the KDIGO (20). AKI was defined as follows: Stage 1, increase in serum creatinine (Scr) 1.5–1.9 times baseline or 0.3 g/dL increase in Scr or urine output <0.5 mL/kg for 6–12 h; Stage 2, increase in Scr 2.0–2.9 times baseline or urine output <0.5 mL/kg for 12 h; and Stage 3, Scr greater than three times baseline or urine output <0.3 mL/kg for 24 h.

**Figure 1:**
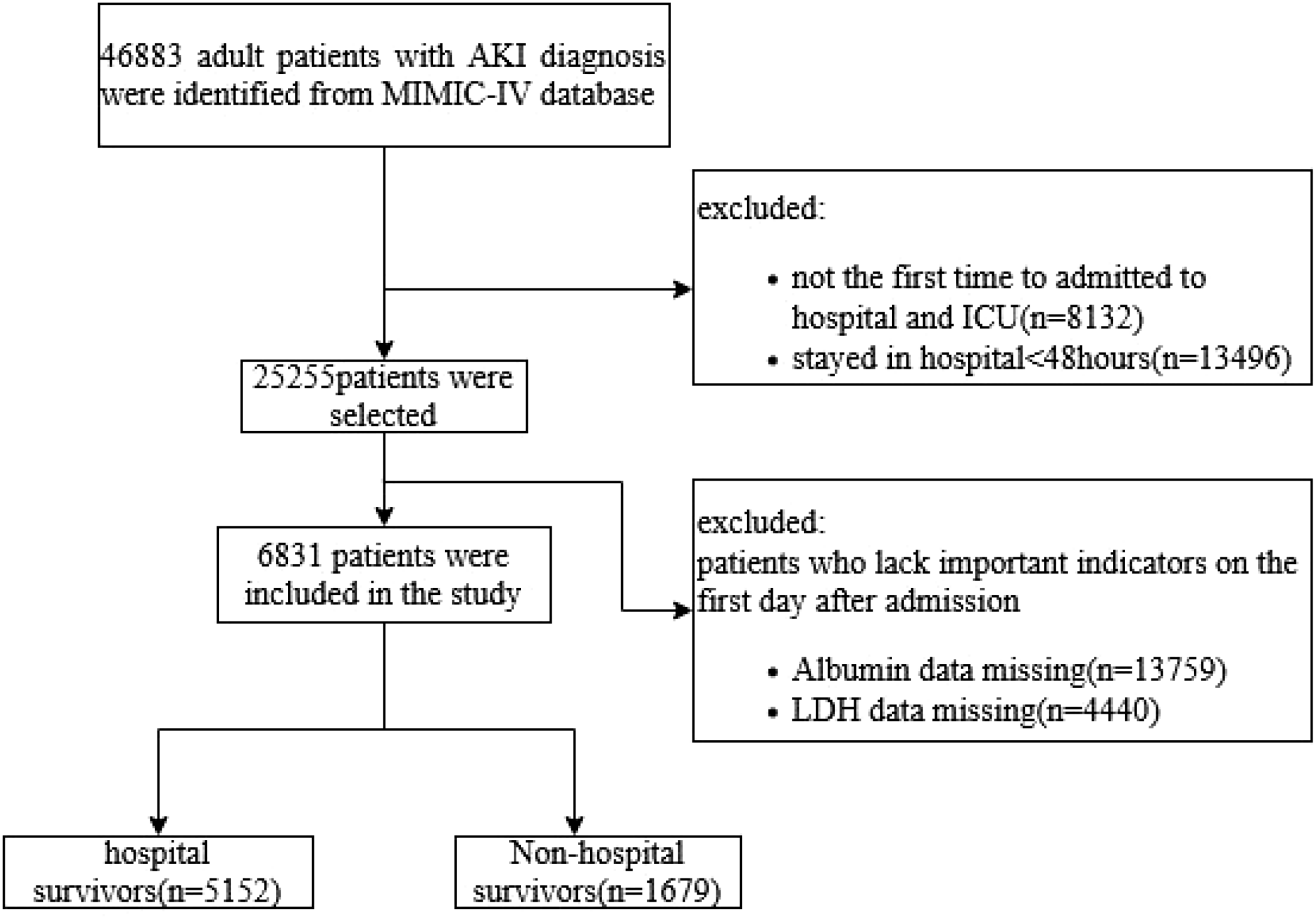
Flowchart of patient selection. AKI, acute kidney injury; LDH, lactate dehydrogenase.

The exclusion criteria were as follows: 1) patients aged <18 years; 2) patients who died within 48 h of ICU admission or stayed in ICU less than 48 h; 3) multiple admissions to the ICU, for whom only data from the first admission were extracted; and 4) patients with missing data for serum albumin or LDH, (**Figure 1**).

### 2.2 Data extraction

In this study, the information was extracted using the Post-gresSQL software (version 13.7.2) and Navicat Premium software (version 16) through the execution of a Structured Query Language (SQL). Comorbidities were determined according to the 9th and 10th Edition Clinical revision codes.

Data extracted from the MIMIC-IV database on the first 24 h of ICU admission included the following:

1. Demographic variables: age, sex, and race.
2. Vital signs: respiratory rate (RR), heart rate, mean arterial pressure (MAP), and oxygen saturation levels (SpO_2_) were recorded on the first day of admission.
3. Comorbidities: myocardial infarction (MI), congestive heart failure (CHF), cerebrovascular disease, chronic obstructive pulmonary disease (COPD), severe liver disease, diabetes, metastatic solid tumor, and chronic kidney disease (CKD).
4. Laboratory tests were performed within the initial 24 h of ICU admission, including white blood cell (WBC) count, platelet count, serum potassium level, hemoglobin level, blood glucose level, serum urea nitrogen level [blood urea nitrogen (BUN)], Scr level, LDH, ALB, and other laboratory markers. If a variable was measured multiple times within the previous 24 h, the mean value was used.
5. Acute Physiology Score III (APSIII), Charlson Comorbidity Index (CCI), and Sequential Organ Failure Assessment (SOFA) score were used to assess the severity of illness upon admission.
6. Medical interventions during hospitalization include the administration of vasoactive drugs and antibiotics, the first 24-h urine output (UO), the use of continuous renal replacement therapy (CRRT) and CRRT mode, and the AKI stage during hospitalization.
7. The duration of ICU stay, the overall length of hospital stay, and LAR were calculated using the LDH (U/L)/ALB (g/L) ratio.

### 2.3 Groups and outcomes

Patients were classified based on survival outcomes during the ICU stay into two groups: the survival group and the death group. To mitigate potential bias between these groups, propensity score matching (PSM) analysis was conducted. The PSM analysis employed a 1:1 nearest-neighbor matching algorithm with a caliper width of 0.05 to ensure close matching of the pairs (**Supplementary Figures S1A, B**).

The primary outcome of the present study was ICU mortality, and the secondary endpoints were in-hospital mortality and within 30 and 90 days after admission to the ICU.

### 2.4 Statistical analysis

Normal distribution was assessed using the Shapiro–Wilk test. Continuous variables were presented as mean ± standard deviation (SD) for variables with normal distribution or as median [interquartile range (IQR)] for variables without normal distribution and compared using either Student’s t-test or the Mann–Whitney U test, respectively. Categorical variables were presented as numbers and percentages (%) and were compared using the chi-square test. LAR was examined as both a continuous variable and a categorical variable. Multivariate Cox proportional hazards models were constructed to study the association between LAR and ICU mortality, and the hazard ratio (HR) of mortality was calculated. In the multiple regression analysis models, adjusted covariates were selected based on the difference in baseline characteristics among the groups in which *p* < 0.05 or *p* < 0.001. Furthermore, restricted cubic spline (RCS) was performed to determine the linear and non-linear relationships between the LAR value and the risk of mortality. Subgroup analysis was performed to verify the robustness of the initial results and to find potential interactive factors.

Data analysis was conducted using the Stata software version 14.0 and the Rstudio programming language. A two-tailed *p*-value of less than 0.05 was considered to indicate statistical significance.

## 3 Results

### 3.1 Population and baseline information

According to the inclusion and exclusion criteria, a total of 6,831 patients with AKI were included in the current study (**Figure 1**). Baseline characteristics of the patients are shown in **Table 1**. The average age of the participants was 64.73 ( ± 16.54) years, and men constituted 57.84% of the study population. It was observed that the death group exhibited higher mean values of BUN, Scr, potassium, LAR level, SOFA score, APSIII score, and CCI (all *p* < 0.001). It also had a higher incidence of sepsis (88.45% vs. 73.37%, *p* < 0.001). The patients in the death group were more likely to have a history of cerebrovascular (19% vs. 16.36%, *p* = 0.013), chronic kidney disease (12.03% vs. 7.01%, *p* < 0.001), severe liver disease (19.24% vs. 11.20%, *p* < 0.001), and solid tumor (11.73% vs. 5.94%, *p* < 0.001). However, no statistical differences were found in the history of MI, CHF, COPD, and diabetes (all *p* > 0.05).

**Table 1:** Baseline characteristics of patients in the survival and death groups before and after propensity score matching (PSM).

Variable	Before PSM						After PSM					
	Total (n = 6,831)	Survival group (n = 5,152)	Death group (n = 1,679)	Statistic	*p*	SMD	Total (n = 1,676)	Survival group (n = 838)	Death group (n = 838)	Statistic	*p*	SMD
Age	64.73 ± 16.54	63.94 ± 16.73	67.15 ± 15.71	t = −7.136	<0.001	0.204	67.55 ± 16.47	67.87 ± 15.91	67.22 ± 17.01	t = 0.810	0.418	−0.038
Gender, n (%)				χ^2^ = 0.027	0.870					χ^2^ = 0.199	0.656	
F	2,880 (42.16)	2,175 (42.22)	705 (41.99)			−0.005	701 (41.83)	346 (41.29)	355 (42.36)			0.022
M	3,951 (57.84)	2,977 (57.78)	974 (58.01)			0.005	975 (58.17)	492 (58.71)	483 (57.64)			−0.022
SOFA	7.02 ± 4.05	6.46 ± 3.80	8.76 ± 4.30	t = −19.585	<0.001	0.536	7.73 ± 4.02	7.66 ± 4.08	7.79 ± 3.95	t = −0.669	0.504	0.033
APSIII	57.56 ± 23.60	53.47 ± 21.43	70.12 ± 25.47	t = −24.146	<0.001	0.654	62.23 ± 23.27	61.97 ± 23.76	62.48 ± 22.79	t = −0.448	0.654	0.022
Charlson Comorbidity Index	5.50 ± 3.07	5.24 ± 3.04	6.31 ± 3.02	t = −12.519	<0.001	0.354	6.24 ± 3.12	6.32 ± 3.18	6.16 ± 3.06	t = 1.064	0.287	−0.053
AKI stage, n (%)				χ^2^ = 460.576	<0.001					χ^2^ = 0.275	0.872	
1	2,933 (42.94)	2,396 (46.51)	537 (31.98)			−0.311	690 (41.17)	349 (41.65)	341 (40.69)			−0.019
2	2,661 (38.95)	2,116 (41.07)	545 (32.46)			−0.184	634 (37.83)	317 (37.83)	317 (37.83)			0.000
3	1,237 (18.11)	640 (12.42)	597 (35.56)			0.483	352 (21)	172 (20.53)	180 (21.48)			0.023
Urine output (mL)	1,255.00 (700.00, 2,099.50)	1,350.00 (795.75, 2,195.00)	970.00 (423.00, 1,677.50)	Z = −14.292	<0.001	−0.351	1,121.50 (600.00, 1,895.00)	1,107.50 (623.00, 1,857.50)	1,141.00 (580.00, 1,925.00)	Z = −0.464	0.642	0.063
Vital signs												
Heart rate (bpm)	88.75 ± 17.96	88.01 ± 17.71	91.02 ± 18.52	t = −5.835	<0.001	0.162	88.77 ± 18.53	88.50 ± 18.62	89.05 ± 18.44	t = −0.609	0.543	0.030
MAP (mmHg)	78.09 ± 10.86	78.75 ± 11.05	76.04 ± 9.98	t = 9.397	<0.001	−0.271	76.81 ± 10.31	76.75 ± 10.45	76.87 ± 10.18	t = −0.235	0.814	0.012
Resp rate (bpm)	20.64 ± 4.40	20.31 ± 4.26	21.65 ± 4.67	t = −10.405	<0.001	0.286	20.78 ± 4.50	20.69 ± 4.56	20.86 ± 4.44	t = −0.752	0.452	0.037
SpO_2_ (%)	97.03 (95.49, 98.48)	97.07 (95.60, 98.50)	96.84 (95.16, 98.45)	Z = −3.660	<0.001	−0.142	97.16 (95.50, 98.64)	97.28 (95.60, 98.64)	97.08 (95.47, 98.63)	Z = −1.106	0.269	−0.042
Laboratory results												
WBC (K/μL)	11.70 (8.40, 16.40)	11.45 (8.30, 15.80)	12.85 (8.90, 18.05)	Z = −6.700	<0.001	0.132	12.05 (8.65, 17.00)	11.62 (8.45, 16.50)	12.47 (8.90, 17.40)	Z = −1.671	0.095	0.008
Platelets (K/μL)	181.50 (119.50, 252.75)	186.00 (127.38, 256.00)	164.00 (98.25, 242.50)	Z = −8.032	<0.001	−0.164	174.00 (111.88, 249.50)	171.50 (115.62, 245.00)	176.50 (107.12, 251.38)	Z = −0.253	0.801	0.011
Hemoglobin (g/dL)	10.60 ± 2.28	10.72 ± 2.26	10.21 ± 2.29	t = 8.097	<0.001	−0.225	10.34 ± 2.28	10.29 ± 2.22	10.38 ± 2.34	t = −0.789	0.430	0.038
Albumin (g/dL)	3.11 ± 0.67	3.18 ± 0.66	2.93 ± 0.69	t = 12.874	<0.001	−0.358	3.02 ± 0.67	3.01 ± 0.66	3.03 ± 0.68	t = −0.663	0.507	0.032
LDH (IU/L)	314.00 (226.00, 509.50)	293.75 (216.00, 455.12)	396.50 (269.25, 684.50)	Z = −16.113	<0.001	0.159	337.00 (235.88, 531.25)	305.25 (219.00, 503.88)	353.75 (254.25, 578.50)	Z = −4.741	<0.001	−0.022
LAR (IU/g)	10.40 (7.09, 17.72)	9.45 (6.69, 15.60)	14.28 (9.03, 25.06)	Z = −18.866	<0.001	0.193	11.44 (7.67, 19.27)	10.37 (7.06, 18.29)	12.16 (8.43, 20.13)	Z = −4.155	<0.001	−0.046
Potassium (mmol/L)	4.50 (4.10, 5.20)	4.50 (4.10, 5.10)	4.60 (4.20, 5.30)	Z = −6.038	<0.001	0.137	4.60 (4.10, 5.20)	4.60 (4.10, 5.20)	4.60 (4.20, 5.20)	Z = −1.069	0.285	0.044
BUN (mg/dL)	25.00 (15.50, 42.25)	23.00 (15.00, 39.12)	31.50 (19.00, 50.50)	Z = −12.630	<0.001	0.283	29.00 (18.00, 47.00)	28.50 (18.00, 48.38)	29.00 (17.50, 46.00)	Z = −0.341	0.733	−0.016
SCr (mg/dL)	1.20 (0.85, 2.00)	1.15 (0.80, 1.90)	1.45 (0.95, 2.35)	Z = −9.759	<0.001	0.130	1.30 (0.90, 2.15)	1.30 (0.90, 2.15)	1.30 (0.90, 2.10)	Z = −0.052	0.959	−0.008
Glucose (mg/dL)	135.20 (111.50, 171.83)	133.16 (110.75, 168.50)	141.57 (113.73, 182.19)	Z = −5.125	<0.001	−0.037	140.87 (112.79, 175.69)	140.26 (114.70, 176.00)	141.49 (111.75, 174.47)	Z = −0.085	0.932	−0.032
Comorbidity disease, n (%)												
Myocardial infarction	1,383 (20.25)	1,037 (20.13)	346 (20.61)	χ^2^ = 0.180	0.671	0.012	350 (20.88)	178 (21.24)	172 (20.53)	χ^2^ = 0.130	0.718	−0.018
Congestive heart failure	2,376 (34.78)	1,787 (34.69)	589 (35.08)	χ^2^ = 0.087	0.768	0.008	618 (36.87)	299 (35.68)	319 (38.07)	χ^2^ = 1.025	0.311	0.049
Chronic obstructive pulmonary disease	1,750 (25.62)	1,305 (25.33)	445 (26.50)	χ^2^ = 0.916	0.339	0.027	448 (26.73)	228 (27.21)	220 (26.25)	χ^2^ = 0.195	0.659	−0.022
Severe liver disease	900 (13.18)	577 (11.20)	323 (19.24)	χ^2^ = 71.523	<0.001	0.204	277 (16.53)	141 (16.83)	136 (16.23)	χ^2^ = 0.108	0.742	−0.016
Diabetes with Cc	761 (11.14)	575 (11.16)	186 (11.08)	χ^2^ = 0.009	0.925	−0.003	200 (11.93)	106 (12.65)	94 (11.22)	χ^2^ = 0.818	0.366	−0.045
Metastatic solid tumor	503 (7.36)	306 (5.94)	197 (11.73)	χ^2^ = 62.315	<0.001	0.180	173 (10.32)	90 (10.74)	83 (9.90)	χ^2^ = 0.316	0.574	−0.028
Cerebrovascular	1,162 (17.01)	843 (16.36)	319 (19.00)	χ^2^ = 6.237	0.013	0.067	321 (19.15)	163 (19.45)	158 (18.85)	χ^2^ = 0.096	0.756	−0.015
Chronic kidney disease	563 (8.24)	361 (7.01)	202 (12.03)	χ^2^ = 42.264	<0.001	0.154	152 (9.07)	79 (9.43)	73 (8.71)	χ^2^ = 0.260	0.610	−0.025
Interventions												
CRRT use, n (%)	411 (6.02)	217 (4.21)	194 (11.55)	χ^2^ = 120.733	<0.001	0.230	129 (7.7)	68 (8.11)	61 (7.28)	χ^2^ = 0.412	0.521	−0.032
Antibiotic use, n (%)	2,569 (37.61)	1,372 (26.63)	1,197 (71.29)	χ^2^ = 1,076.489	<0.001	0.987	890 (53.1)	437 (52.15)	453 (54.06)	χ^2^ = 0.613	0.434	0.038
Vasopressor use, n (%)	3,380 (49.48)	2,289 (44.43)	1,091 (64.98)	χ^2^ = 213.927	<0.001	0.431	955 (56.98)	475 (56.68)	480 (57.28)	χ^2^ = 0.061	0.805	0.012
Ventilation status, n (%)				χ^2^ = 1,908.994	<0.001					χ^2^ = 0.963	0.966	
HFNC	67 (1.06)	32 (0.68)	35 (2.15)			0.101	37 (2.21)	19 (2.27)	18 (2.15)			−0.008
InvasiveVent	1,593 (25.17)	535 (11.38)	1,058 (64.99)			1.124	667 (39.8)	333 (39.74)	334 (39.86)			0.002
None	6 (0.09)	5 (0.11)	1 (0.06)			−0.018	3 (0.18)	2 (0.24)	1 (0.12)			−0.035
NonInvasiveVent	40 (0.63)	31 (0.66)	9 (0.55)			−0.014	10 (0.6)	4 (0.48)	6 (0.72)			0.028
Supplemental Oxygen	4,424 (69.91)	3,909 (83.17)	515 (31.63)			−1.108	939 (56.03)	469 (55.97)	470 (56.09)			0.002
Tracheostomy	198 (3.13)	188 (4.00)	10 (0.61)			−0.433	20 (1.19)	11 (1.31)	9 (1.07)			−0.023
Sepsis, n (%)	5,265 (77.08)	3,780 (73.37)	1,485 (88.45)	χ^2^ = 162.888	<0.001	0.472	1,396 (83.29)	700 (83.53)	696 (83.05)	χ^2^ = 0.069	0.793	−0.013
ICU LOS	4.96 (3.12, 9.17)	4.77 (3.04, 8.78)	5.81 (3.61, 10.87)	Z = −7.684	<0.001	0.116	5.26 (3.31, 10.06)	5.12 (3.21, 10.16)	5.46 (3.50, 10.03)	Z = −0.771	0.441	−0.045
Hospital LOS	11.85 (7.05, 20.61)	12.82 (7.88, 21.76)	8.96 (4.77, 16.55)	Z = −16.241	<0.001	−0.345	11.85 (6.63, 20.16)	12.67 (7.77, 20.99)	10.82 (5.31, 18.95)	Z = −4.693	<0.001	−0.055

Data are presented as the mean ± SD or median (IQR) for skewed variables or numbers (proportions) for categorical variables.

SOFA, Sequential Organ Failure Assessment; APSIII, Acute Physiology Score III; MAP, mean arterial pressure; SpO_2_, oxygen saturation levels; BUN, blood urea nitrogen; Scr, serum creatinine; WBC, white blood cell; LDH, lactate dehydrogenase; LAR lactate dehydrogenase to albumin ratio; AKI, acute kidney injury; UO, urine output; CRRT, continuous renal replacement therapy; HFNC, high-flow nasal cannula oxygen therapy; ICU, intensive care unit; ICU LOS, length of intensive care unit stay; Hospital LOS, length of hospital stay; SMD, Standardized Mean Difference.

The survivors typically had shorter ICU stays (4.77 vs. 5.81, *p* < 0.001); less use of CRRT (4.21% vs. 11.55%, *p* < 0.001), antibiotics (26.63% vs. 71.29%, *p* < 0.001), and vasopressors (44.43% vs. 64.98%, *p* < 0.001); more urine output (1,350 vs. 970 mL, *p* < 0.001); and significantly higher levels of albumin, platelets, and hemoglobin.

After PSM, a total of 838 pairs were successfully matched, achieving well-balanced baseline demographic characteristics between groups. It was observed that the death group also exhibited higher values of LAR (12.16 vs. 10.37, *p* < 0.001).

### 3.2 Association between LAR and mortality

**Figure 2:**
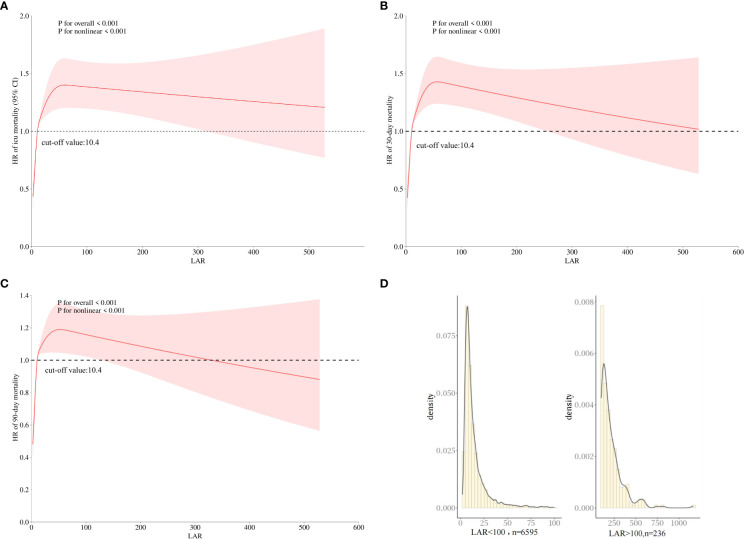
The association between LAR and the HR of ICU **(A)** and 30-day **(B)** and 90-day **(C)** mortality using restricted cubic spline analysis. **(D)** The distribution of LAR values. HR, hazard ratio; LAR, lactate dehydrogenase to albumin ratio; ICU, intensive care unit.

A restricted cubic spline curve was employed in order to flexibly visualize and analyze the association between LAR and ICU mortality in patients with AKI in **Figure 2A** (*p* for non-linear <0.001).

In **Figure 2A**, when LAR is less than 10.4 IU/g, HR is less than 1. When LAR is greater than 10.4, HR is greater than 1, and LAR is positively correlated with HR. In addition, similar linear associations were also observed in the analysis of 30-day mortality and 90-day mortality (**Figures 2B, C**). Therefore, a LAR of 10.4 was used as the cutoff point to generate the high-LAR and low-LAR subgroups for the subgroup analysis. Although an elevated LAR > 100 was inversely associated with mortality, this finding was observed only in a very small subset of samples. **Figure 2D** shows the distribution of LAR values, which indicates that the majority of the cohort (over 97%) fell within the 0–100 range, suggesting limited reliability of the association at extreme LAR levels.

**Figure 3:**
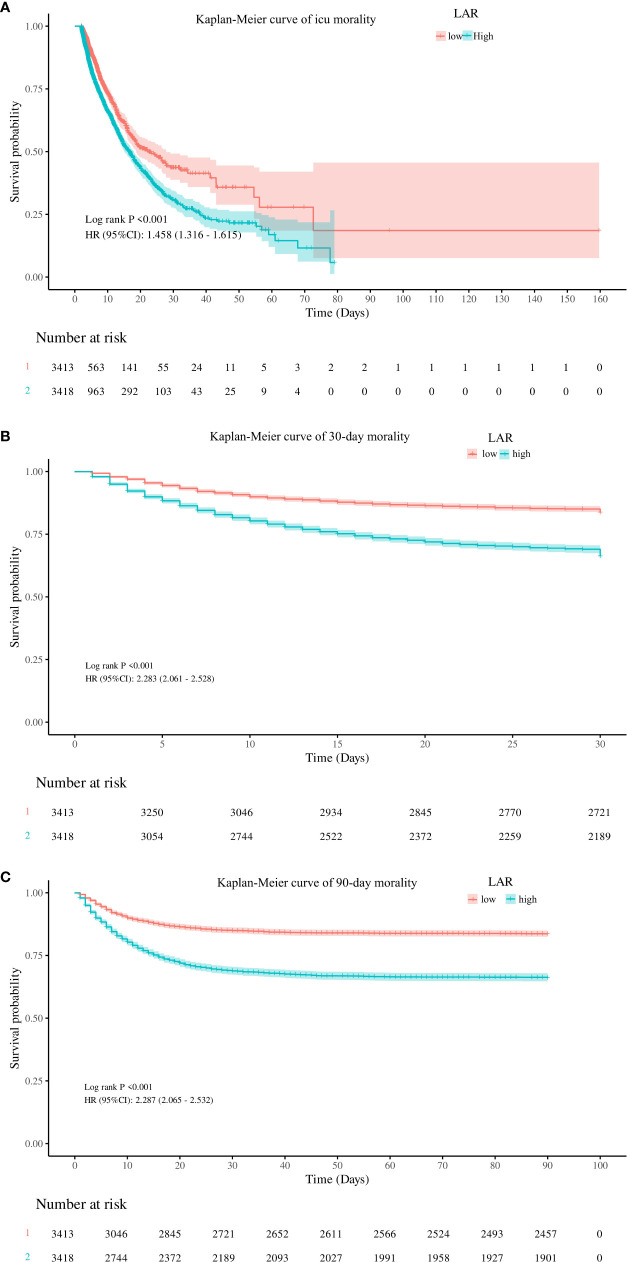
Kaplan–Meier curves for ICU stay **(A)** and 30-day **(B)** and 90-day **(C)** accumulative survival rates stratified by the high and low groups of LAR. ICU, intensive care unit; LAR, lactate dehydrogenase to albumin ratio.

To evaluate cumulative survival at different levels of LAR, we generated survival curves for patients with AKI by stratifying based on the high and low LAR levels. The Kaplan–Meier analysis showed that patients with lower LAR levels had a significantly higher ICU survival probability (*p* < 0.001) (**Figure 3A**). In addition, similar results were observed in the 30-day and 90-day survival curves (**Figures 3B, C**).

### 3.3 LAR categories and clinical outcomes

Patients were further divided into four categories according to the IQR of the LAR value (Q1–Q4 categories). The crude outcomes of those four subgroups are shown in **Table 2**. **Table 2** reveals a gradual escalation in SOFA and APSIII scores across the Q1 to Q4 subgroups, and all pairwise comparisons demonstrated statistical significance (*p* < 0.001). Regarding clinical outcomes, the Q1 to Q4 subgroups exhibited an increasing trend in the proportion of CRRT utilization, ICU length of stay (LOS), and hospital LOS. CRRT utilization increased from 2.69% to 11.59%, ICU LOS extended from 4.13 (2.88, 7.22) days to 6.12 (3.56, 11.74) days, and hospital LOS rose from 10.02 (6.58, 16.74) days to 13.99 (7.50, 23.07) days (*p* for trend <0.001). Mortality rates in the Q4 group were markedly elevated (in-hospital, 38.41%; 30 days, 40.69%; 90 days, 48.24%) compared to lower quartiles (all *p* < 0.001).

**Table 2:** Comparisons of the clinical outcomes among patients in different LAR categories.

Variables		Q1 (LAR ≤ 7.09)	Q2 (7.09 < LAR ≤ 10.4)	Q3 (10.4 < LAR ≤ 17.72)	Q4 (LAR > 17.72)	Statistic	*p*
Number (n)	6,831	1,708	1,705	1,710	1,708		NA
Age	64.73 ± 16.54	66.16 ± 16.14	66.82 ± 16.20	64.89 ± 16.11	61.05 ± 17.10	F = 42.36	<0.001
SOFA	7.02 ± 4.05	5.40 ± 3.55	6.52 ± 3.71	7.36 ± 3.88	8.81 ± 4.26	F = 237.97	<0.001
APSIII	57.56 ± 23.60	48.25 ± 20.02	54.26 ± 21.24	59.17 ± 22.75	68.54 ± 25.27	F = 250.29	<0.001
Charlson comorbidity index	5.50 ± 3.07	5.38 ± 2.94	5.76 ± 3.00	5.54 ± 3.07	5.34 ± 3.24	F = 6.60	<0.001
Urine output	1,255.00 (700.00, 2,099.50)	1,355.00 (813.00, 2,157.50)	1,325.00 (765.00, 2,160.00)	1,257.50 (688.00, 2,075.00)	1,060.00 (490.75, 1,920.50)	χ^2^ = 79.78#	<0.001
Potassium	4.50 (4.10, 5.20)	4.40 (4.00, 4.90)	4.50 (4.10, 5.00)	4.60 (4.10, 5.10)	4.80 (4.30, 5.60)	χ^2^ = 271.05#	<0.001
ALB	3.10 (2.65, 3.60)	3.60 (3.20, 3.95)	3.20 (2.80, 3.60)	2.90 (2.50, 3.40)	2.80 (2.30, 3.30)	χ^2^ = 1,148.85#	<0.001
BUN	25.00 (15.50, 42.25)	20.50 (13.50, 34.50)	24.00 (15.00, 41.00)	25.50 (16.00, 44.50)	30.00 (19.00, 48.12)	χ^2^ = 179.97#	<0.001
Scr	1.20 (0.85, 2.00)	1.05 (0.75, 1.65)	1.15 (0.80, 1.85)	1.25 (0.80, 2.00)	1.55 (1.00, 2.55)	χ^2^ = 212.89#	<0.001
AKI stage, n (%)						χ^2^ = 216.46	<0.001
1	2,933 (42.94)	719 (42.10)	762 (44.69)	780 (45.61)	672 (39.34)		
2	2,661 (38.95)	756 (44.26)	712 (41.76)	660 (38.60)	533 (31.21)		
3	1,237 (18.11)	233 (13.64)	231 (13.55)	270 (15.79)	503 (29.45)		
CRRT use, n (%)	411 (6.02)	46 (2.69)	63 (3.70)	104 (6.08)	198 (11.59)	χ^2^ = 143.54	<0.001
CRRT mode, n (%)						-	0.727
CVVH	12 (2.92)	2 (4.35)	3 (4.76)	1 (0.96)	6 (3.03)		
CVVHD	7 (1.70)	0 (0.00)	1 (1.59)	2 (1.92)	4 (2.02)		
CVVHDF	392 (95.38)	44 (95.65)	59 (93.65)	101 (97.12)	188 (94.95)		
ICU LOS	4.96 (3.12, 9.17)	4.13 (2.88, 7.22)	4.60 (3.02, 7.79)	5.46 (3.34, 10.03)	6.12 (3.56, 11.74)	χ^2^ = 210.59#	<0.001
Hospital LOS	11.85 (7.05, 20.61)	10.02 (6.58, 16.74)	11.16 (6.99, 18.87)	12.92 (7.85, 22.14)	13.99 (7.50, 23.07)	χ^2^ = 106.39#	<0.001
Hospital mortality (%)	1,679 (24.58)	212 (12.41)	336 (19.71)	475 (27.78)	656 (38.41)	χ^2^ = 343.85	<0.001
30-day mortality, (%)	1,921 (28.12)	277 (16.22)	415 (24.34)	534 (31.23)	695 (40.69)	χ^2^ = 273.46	<0.001
90-day mortality, (%)	2,473 (36.20)	387 (22.66)	569 (33.37)	693 (40.53)	824 (48.24)	χ^2^ = 262.64	<0.001

LAR, lactate dehydrogenase to albumin ratio; SOFA, Sequential Organ Failure Assessment; APSIII, Acute Physiology Score III; ALB, albumin; BUN, blood urea nitrogen; Scr, serum creatinine; AKI, acute kidney injury; CRRT, continuous renal replacement therapy; ICU LOS, length of intensive care unit stay; Hospital LOS, length of hospital stay; CVVH, Continuous Veno-Venous Hemofiltration; CVVHD, Continuous Veno-Venous Hemodialysis; CVVHDF, Continuous Veno-Venous Hemodiafiltration.

After performing a multi-factor logistic regression analysis (**Supplementary Table S1**), we developed and used four multivariable models to identify significant correlations between the different LAR categories and hospital mortality. **Table 3** displays the HRs and 95% confidence intervals (CIs) for the models. Crude model was without any adjustments. Model 1 was adjusted for age, gender, and race; Model 2 was built upon Model 1 and further adjusted for APSIII, CCI, and comorbidities including MI, CHF, cerebrovascular disease, severe liver disease, and solid tumor. Model 3 encompassed all covariates from Model 2 and also included adjustments for the laboratory tests, first 24-h urine output, and interventions such as antibiotics, vasopressors, and CRRT use.

**Table 3:** Cox proportional hazards regression analysis of LAR categories and clinical outcomes in patients with AKI.

Variables	Unadjusted		Model 1		Model 2			Model 3	
	HR (95% CI)	*p*	HR (95% CI)	*P*	HR (95% CI)		*p*	HR (95% CI)	*p*
Hospital mortality									
LAR	1.01 (1.01–1.01)	<0.001	1.00 (1.00–1.00)	0.450	1.00 (1.00–1.00)		0.302	1.00 (1.00–1.00)	0.428
Q1 (LAR ≤ 7.09)	1.00 (Ref)		1.00 (Ref)		1.00 (Ref)			1.00 (Ref)	
Q2 (7.09 < LAR ≤ 10.4)	1.46 (1.23–1.73)	<0.001	1.43 (1.20–1.70)	<0.001	1.31 (1.10–1.55)	0.003	1.31 (1.10–1.55)	1.30 (1.09–1.55)	0.003
Q3 (10.4 < LAR ≤ 17.72)	1.84 (1.57–2.17)	<0.001	1.87 (1.59–2.20)	<0.001	1.59 (1.35–1.88)	<0.001	1.59 (1.35–1.88)	1.51 (1.27–1.78)	<0.001
Q4 (LAR > 17.72)	2.47 (2.12–2.89)	<0.001	2.68 (2.29–3.13)	<0.001	2.03 (1.73–2.39)	<0.001	2.03 (1.73–2.39)	1.75 (1.49–2.07)	<0.001
30-day mortality									
Q1 (LAR ≤ 7.09)	1.00 (Ref)		1.00 (Ref)		1.00 (Ref)			1.00 (Ref)	
Q2 (7.09 < LAR ≤ 10.4)	1.56 (1.34–1.82)	<0.001	1.54 (1.33–1.80)	<0.001	1.35 (1.16–1.57)		<0.001	1.38 (1.18–1.60)	<0.001
Q3 (10.4 < LAR ≤ 17.72)	2.09 (1.81–2.42)	<0.001	2.17 (1.87–2.50)	<0.001	1.70 (1.47–1.97)		<0.001	1.64 (1.41–1.90)	<0.001
Q4 (LAR > 17.72)	2.96 (2.57–3.40)	<0.001	3.31 (2.87–3.80)	<0.001	2.20 (1.89–2.55)		<0.001	1.97 (1.69–2.29)	<0.001
90-day mortality									
Q1 (LAR ≤ 7.09)	1.00 (Ref)		1.00 (Ref)		1.00 (Ref)			1.00 (Ref)	
Q2 (7.09 < LAR ≤ 10.4)	1.57 (1.38–1.78)	<0.001	1.55 (1.36–1.76)	<0.001	1.37 (1.20–1.56)	<0.001	<0.001	1.39 (1.22–1.58)	<0.001
Q3 (10.4 < LAR ≤ 17.72)	2.01 (1.78–2.28)	<0.001	2.10 (1.85–2.38)	<0.001	1.69 (1.48–1.91)	<0.001	<0.001	1.65 (1.45–1.88)	<0.001
Q4 (LAR > 17.72)	2.64 (2.34–2.98)	<0.001	3.00 (2.65–3.38)	<0.001	2.07 (1.82–2.36)	<0.001	<0.001	1.92 (1.68–2.19)	<0.001

Note. Crude model: adjusted for nothing.

Model 1: Adjusted for age, race, and gender.

Model 2: Model 1+Adjust: APSIII, CCI, MI, CHF, cerebrovascular disease, metastatic solid tumor, and severe liver disease.

Model 3: Model 2+Adjust: antibiotic use, vasopressor use, CRRT use, WBC, Hb, PLT, BUN, Scr, and first 24-h urine output.

HR, hazard ratio; CI, confidence interval; SOFA, Sequential Organ Failure Assessment; APSIII, Acute Physiology Score III; CCI, Charlson Comorbidity Index; MI, myocardial infarction; CHF, congestive heart failure; CKD, chronic kidney disease; WBC, serum white blood cell; Hb, hemoglobin; BUN, blood urea nitrogen; Scr, serum creatinine; UO, urine output; CRRT, continuous renal replacement therapy; PLT, platelet.

**Table 3** shows that when LAR was a continuous variable in one original model, each IU/g increase in LAR was significantly associated with a 1% increase in in-hospital mortality (HR, 1.001, *p* < 0.001) but lost statistical significance in adjusted models (*p* = 0.45 in Model 1, *p* = 0.30 in Model 2, and *p* = 0.43 in Model 3).

In addition, when the LAR was used as a categorical variable (categorized by quartiles), elevated LAR was identified as an independent risk factor for 30-day and in-hospital mortality in all models (all HR > 1, *p* < 0.001). The risk of mortality tended to increase steadily with increasing LAR levels from Q2 (HR, 1.46, 95% CI 1.23–1.73, *p* < 0.001 for in-hospital mortality; HR, 1.56, 95% CI 1.34–1.83, *p* < 0.001 for 30-day mortality) to Q4 (HR, 2.47, 95% CI 2.12–2.89, *p* < 0.001 for in-hospital mortality; HR, 2.96, 95% CI 2.57–3.40, *p* < 0.001 for 30-day mortality) in unadjusted model, with the Q1 category (LAR ≤ 7.09 IU/g) as a reference.

After controlling for age, sex, race, APSIII, CCI, comorbidities, laboratory tests and interventions, the adjusted HRs for the Q2–Q4 groups compared to Q1 were 1.30 (HR, 1.30, 95% CI 1.09–1.55, *p* < 0.001), 1.51 (HR, 1.51, 95% CI 1.27–1.78, *p* < 0.001), and 1.75 (HR, 1.75, 95% CI 1.49–2.07, *p* < 0.001). Investigation of mortality after 30 and 90 days yielded comparable findings.

### 3.4 Receiver operating characteristic analysis

**Figure 4:**
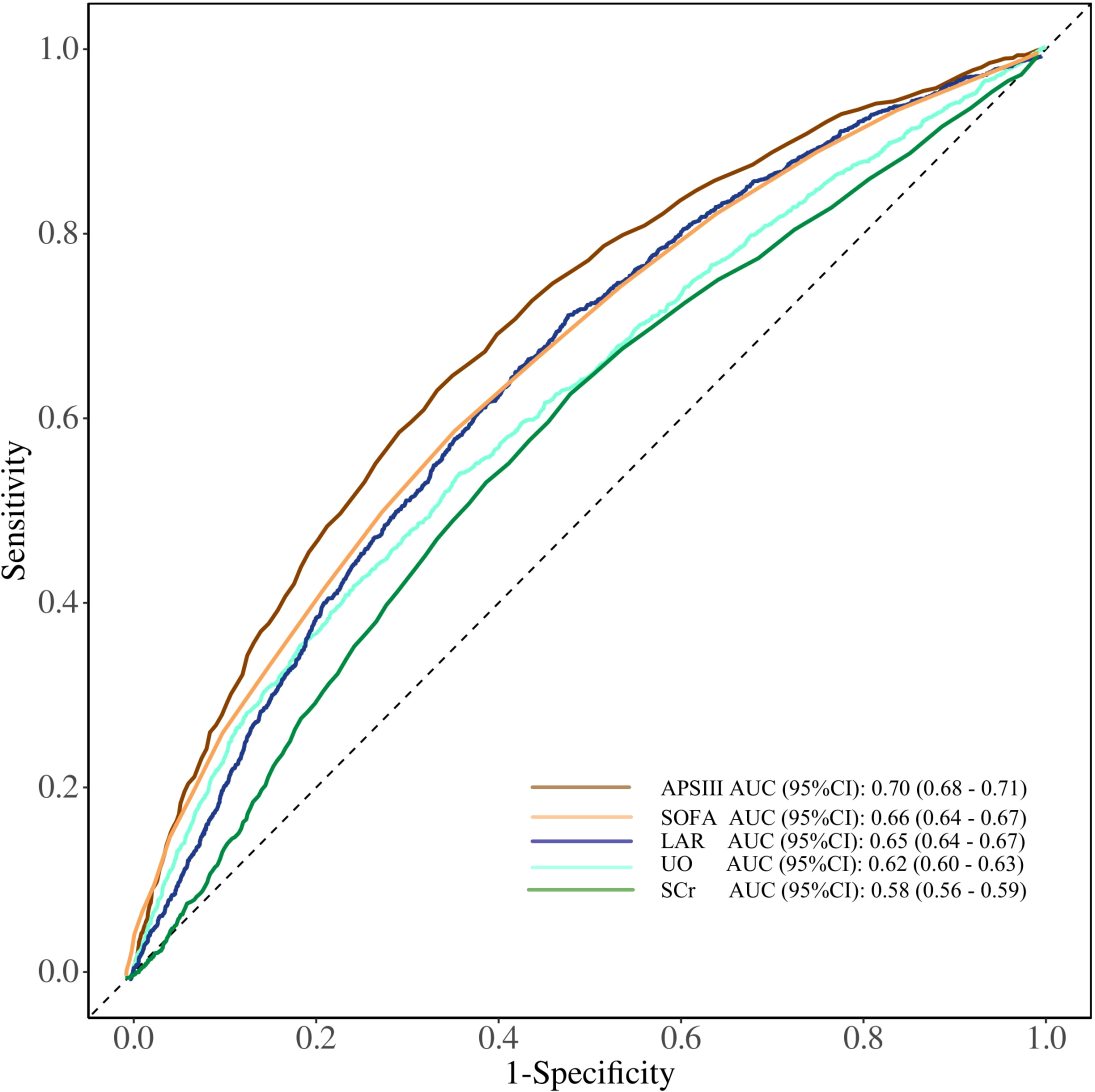
ROC curves of LDH/ALB ratio, SOFA, APSIII score, UO, and Scr for predicting ICU mortality in patients with AKI. SOFA, Sequential Organ Failure Assessment; APSIII, Acute Physiology Score III; UO, urine output; Scr, serum creatinine; LDH/ALB ratio, lactate dehydrogenase to albumin ratio; ROC, receiver operating characteristic; ICU, intensive care unit.

The clinical value of LAR in predicting mortality was determined by receiver operating characteristic (ROC) curve analysis. As shown in **Figure 4**, LAR showed a moderate predictive ability for ICU mortality [area under the curve (AUC) 0.65, 95% CI 0.64–0.67]. LAR had a better predictive ability than urine output (AUC 0.62, 95% CI 0.60–0.63) and Scr (AUC 0.58, 95% CI 0.56–0.59), but less than APSIII (AUC 0.70, 95% CI 0.68–0.71) and SOFA (AUC 0.66, 95% CI 0.64–0.67).

### 3.5 Subgroup analysis and *post-hoc* analysis

**Figure 5:**
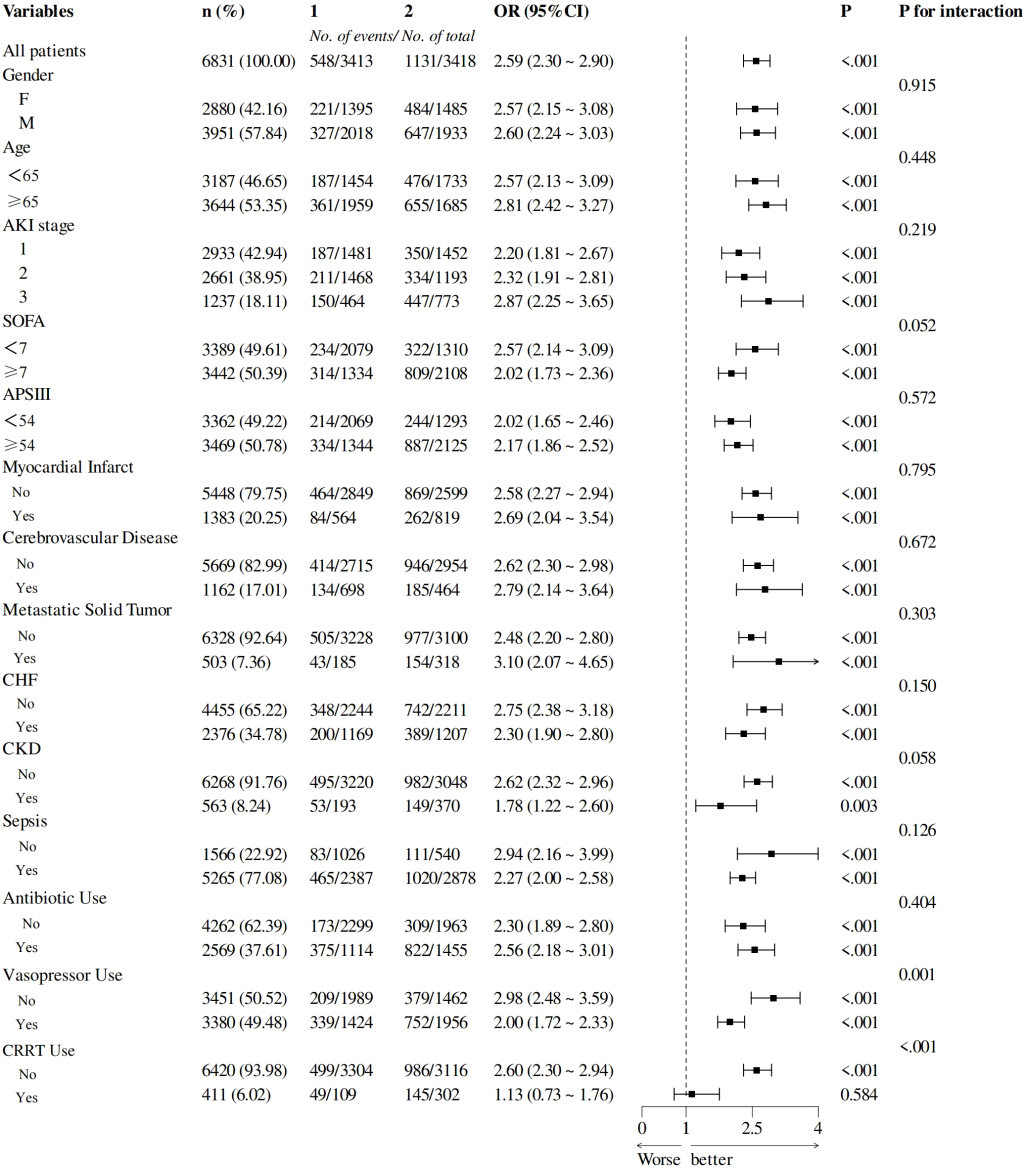
Subgroup analyses of the LDH/ALB ratio in patients with AKI. LDH/ALB, lactate dehydrogenase/albumin; AKI, acute kidney injury.

To explore the differences in the predictive value of LAR among different subgroups, we performed subgroup analysis as shown in **Figure 5**. The higher LAR was consistently associated with increased hospital mortality in different subgroups, including AKI Stage 1 to 3, age <65 or ≥65 years, SOFA <7 or ≥7, APSIII<54 or ≥54, female or male, with or without MI, CHF, solid tumor, cerebrovascular disease, sepsis, CKD, and use of antibiotics.

There was an interaction between the use of vasopressors, CRRT, and LAR on hospital mortality (*p* for interaction = 0.001, <0.001), and the predictive value of LAR was less pronounced in these patients.

**Figure 6:**
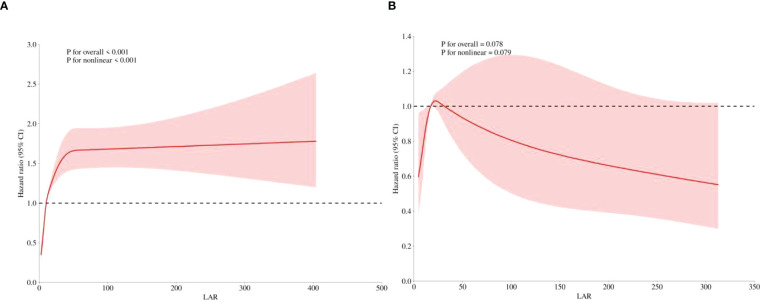
*Post-hoc* analysis. Restricted cubic spline showed the association between the LAR and the hospital mortality was further investigated in the non-CRRT **(A)** and CRRT cohorts **(B)** of patients with AKI. LAR, lactate dehydrogenase to albumin ratio; CRRT, continuous renal replacement therapy; AKI, acute kidney injury.

In the *post-hoc* analysis, the relationship and predictive value between LAR and hospital mortality in patients with or without CRRT use were further determined. **Supplementary Figure S2** illustrates the distribution of LAR across different subgroups. **Figure 6a** shows that a significant positive linear relationship between the LAR and hospital mortality still existed in patients without CRRT use (*p* for non-linear <0.001). However, it did not hold true for AKI patients with CRRT use. As shown in **Figure 6b**, there was no significant trend between LAR and hospital mortality in AKI patients’ CRRT use (*p* for non-linear = 0.079).

## 4 Discussion

### 4.1 Key findings

In this retrospective cohort study, we utilized data from the large-scale MIMIC-IV database to explore the association between the LDH/ALB ratio and the risk of death in AKI patients admitted to the ICU. The current study’s findings suggested that higher levels of the LDH/ALB ratio are associated with increased mortality in critically ill patients with AKI. Notably, even after accounting for possible confounding variables, this association is still statistically significant. Multivariate regression analyses exhibited the independent prognostic significance of the LDH/ALB ratio for ICU all-cause mortality; this association was constant across subgroup analyses and sensitivity analyses, demonstrating the robustness of the finding. Additionally, the current study also highlights the significant association of the LDH/ALB ratio with in-hospital mortality, length of ICU, and 90-day mortality among critically ill patients with AKI.

### 4.2 Relation with previous evidence

Even though the pathogenesis of AKI is still not well recognized, it is often a complex condition regularly implicated by hemodynamic instability, sepsis, and medication toxicity. Regardless of the specific cause, some distinct pathophysiologic processes, including endothelial dysfunction, oxidative stress (OS), changes in microcirculation, and intrarenal inflammation, occur concurrently and in order (21, 22). Inflammation is a major contributor to AKI pathophysiology. Septic shock can cause inflammatory responses inducing AKI and a chronic microinflammatory state, and oxidative stress also exists in diabetes mellitus, chronic kidney disease, and malignancy mediated by inflammatory cytokines. Inflammatory factors and oxidative stress can directly promote apoptosis and further aggravate renal impairment. LDH, as a marker of inflammatory factors and oxidative stress, is involved in the apoptosis of renal tubular epithelial cells. In certain malignancies, elevated levels of LDH correlate with disease activity, tumor proliferation rate, and cellular breakdown, and thus, the combination of these mechanisms may render an individual more susceptible to AKI. It can be speculated that the effects of inflammation, oxidative stress, and apoptosis induced by certain diseases themselves may lead to an enhanced association between LAR and the development of AKI.

Albumin is an indicator of inflammation and systemic nutritional status, studies have shown that albumin has a renoprotective mechanism, and hypoproteinemia is considered an independent risk factor for the prognosis of AKI (23–25). In addition, there is a negative relationship between albumin levels and levels of neutrophil gelatinase-associated lipocalin (NGAL), which is commonly associated with inflammation and kidney injury and is recognized as a biomarker for renal function and damage (26, 27). LDH is not only a metabolite but also a prognostic biomarker for immune surveillance. Renal tissue damage caused by inflammation, oxidative stress, and ischemic/hypoxic events during AKI may lead to the release of intracellular LDH into the serum (7). LDH has been shown to be associated with in-hospital mortality in critically ill patients with acute kidney injury (28). In our present study, we found that patients in the death group had higher LDH and lower albumin than those in the survival group.

The LDH/ALB ratio, which combines factors of oxidative stress, inflammation, and nutritional status, may provide more comprehensive prognostic information than the separate predictive values of LDH or albumin. Recent studies have investigated the clinical value of LDH in combination with albumin. Guan XY et al. (16), in a study of individuals with sepsis, suggested a non-linear relationship between the LDH/ALB ratio and the risk of ICU mortality and indicated that an elevated LDH/ALB ratio (≥10.57) was a significant predictor of all-cause mortality among ICU patients with sepsis. In another study of sepsis, Fang XP, et al. (17) found a positive linear correlation between LAR and the development of sepsis-associated AKI (SA-AKI), and they indicated that LAR 12 h before and after the diagnosis of sepsis is an independent risk factor for the development of SA-AKI in patients with sepsis.

Our study showed that LAR ≥ 10.4 was a significant predictor of all-cause mortality among ICU patients with AKI. To mitigate the effects of heart and liver tissue injuries on the predictive accuracy of LAR, we controlled for several variables including coronary heart disease, heart failure, severe liver disease, solid tumor, and cerebrovascular disorders evaluated in our multi-factor logistic regression analysis. Nevertheless, elevated LAR was identified as an independent risk factor for 30-day, 90-day, and in-hospital mortality in both crude and adjusted models.

Urine output and creatinine are traditional indicators reflecting renal function and can also be used to predict the prognosis of AKI patients (29). Our study further revealed that in predicting in-ICU mortality among AKI patients, LAR demonstrated a better predictive ability than urine output and creatinine by ROC curve analysis, ranking second only to SOFA and APS III scores. This finding further confirms that LAR can serve as a reliable biomarker for predicting AKI prognosis.

In our study, patients were stratified based on sepsis status, and analyses were performed in subgroup analysis, which showed that whether sepsis was present or not, LAR still demonstrated a good correlation with the prognosis of patients with kidney injury. It may be attributed to the fact that a majority of patients with kidney injury experience protein leakage as well as ischemia and hypoxia. Albumin, serving as a critical component in maintaining plasma colloid osmotic pressure, plays a pivotal role in regulating fluid balance and tissue perfusion, thereby mitigating the progression of acute kidney injury (23).

In our subgroup analysis, the predictive effect of LAR on in-hospital mortality showed no significant interaction with the presence of CKD, which contrasts with previous findings. Specifically, in the study by Deng et al. (30), the prognostic value of the LDH/ALB ratio was more pronounced in the non-CKD patient subgroup. Interestingly, in studies investigating the predictive value of the LDH/ALB ratio for sepsis-associated acute kidney injury (SAKI), Fang et al. (17) found that the LDH/ALB ratio lost its prognostic utility for SAKI progression in patients with CKD, whereas it remained significant in non-CKD patients. They proposed that this discrepancy may be attributed to podocyte injury-induced proteinuria, which substantially reduces serum albumin levels and reduces the physiological reserve of LDH in the cell. In our study, even though the LDH/ALB ratio demonstrated a more pronounced predictive effect on in-hospital mortality in non-CKD patients compared to the CKD group (OR 2.62 vs. 1.78), this difference did not reach statistical significance (*p* for interaction = 0.058).

Furthermore, our subgroup analysis showed that there was an interaction among the use of vasopressors, CRRT, and LAR on hospital mortality, and in *post-hoc* analysis, the association between LAR and in-hospital mortality was further examined in AKI patients with and without CRRT using restricted cubic splines (RCS). The prognostic value of LAR on hospital mortality was still robust in the non-CRRT subgroup but disappeared in the CRRT subgroup. We speculated that the possible mechanism may be due to the reduction of serum albumin levels, which diminishes the predictive power of the LDH/ALB ratio. Our findings serve as a reminder that CRRT use should not be ignored when using the LDH/ALB ratio to predict mortality.

### 4.3 Strengths and limitations

This research possesses several notable strengths. First, the data utilized in this study were obtained from the well-established MIMIC-IV 3.0 database, which was a high-quality intensive care database with a substantial sample size, enhancing the accuracy and reliability of our findings. The MIMIC-IV database version 3.0 was updated in July 2024 with three additional years of patient data (2020–2022). While the number of ICU admissions increased by less than 30%, the corresponding chart event table volume grew by nearly 50%, indicating that the newly added patient data exhibit finer granularity compared to previous records. Additionally, we employed rigorous statistical techniques, including PSM analysis and detailed subgroup analyses, to avoid the potential influence of confounding factors on the outcomes of our study.

This study had several limitations. First, this is a retrospective cohort study, and the lack of information regarding AKI etiologies in the database restricts the comprehensiveness and level of detail in the study. Therefore, we cannot accurately determine the time of all AKI occurrences, which may affect our accurate assessment of the relationship between the LDH/ALB ratio and the time of AKI occurrence.

Second, we excluded some participants with missing LDH and albumin data, which may lead to selection bias and limit the generalizability of the study results. There was an absence of data regarding interventions during the initial stabilization phase; for instance, the administration of albumin could potentially result in reduced LAR levels and improved survival outcomes, representing an unmeasured confounding variable, which may have a certain impact on the promotion of the study conclusions.

Third, we only extracted data on admission, and we could not detect the fluctuations of LDH or albumin, and inflammatory and immune response indicators during hospitalization. As dynamic change indicators, their values at a single point in time may not accurately reflect the true situation of patients. The absence of this information may limit our comprehensive understanding of the overall condition of the patient, thereby affecting the in-depth analysis of the relationship between the LAR and the occurrence of AKI. Trajectory analysis to identify the different trajectory groups of LAR would be an optional method to solve this problem. Future studies are needed to address these issues, which will be of high value.

## 5 Conclusion

Our research suggests that LAR monitoring may be promising as a prognostic marker among patients with AKI. Higher LAR is associated with greater ICU mortality.

## Data Availability

The datasets presented in this study can be found in online repositories. The names of the repository/repositories and accession number(s) can be found below: https://physionet.org/content/mimiciv.

## Supplementary material

Supplementary Figure/Image S1 and S2 Showed The PSM analysis which employed a 1:1 nearest-neighbor matching algorithm with a caliper width of 0.05 to ensure close matching of the pairs.

Supplementary Figure/Image S3 Showed the distribution of LAR across different subgroups (1 represents the crrt group and 0 represents the non-crrt group).

SUPPLEMENTARY TABLE 1 A multi-factor logistic regression analysis.
