# Supplementary figures and images for: Association between lactate dehydrogenase to albumin ratio and ICU mortality in patients with acute kidney injury: a retrospective cohort study
Source: Front Nephrol. 2025 Jun 2;5:1583913. doi: 10.3389/fneph.2025.1583913 (PMC12171449; doi:10.3389/fneph.2025.1583913)

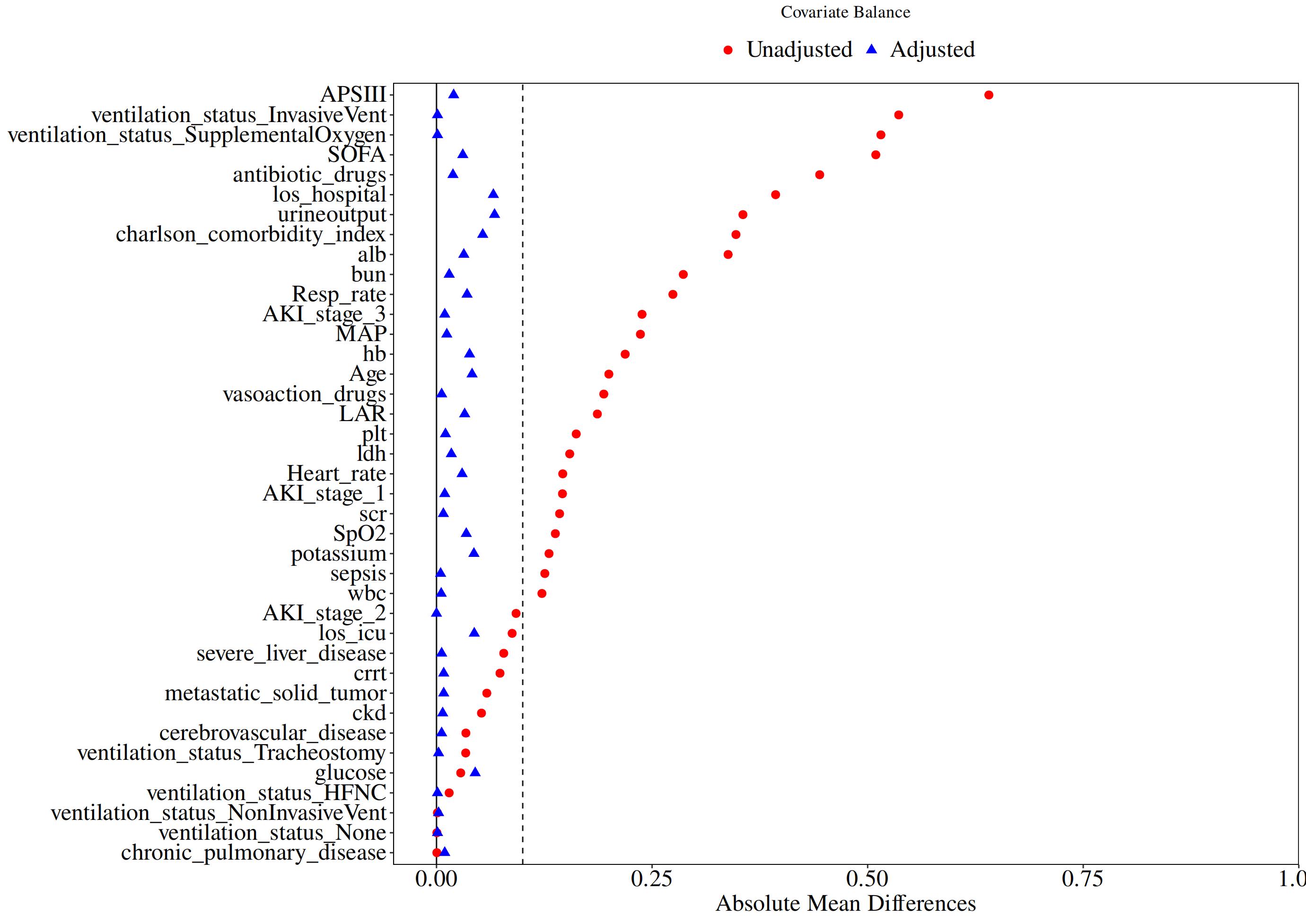

Supplement: Supplementary Figure/Image S1 and S2 — Showed The PSM analysis which employed a 1:1 nearest-neighbor matching algorithm with a caliper width of 0.05 to ensure close matching of the pairs. [file Image1.jpeg]

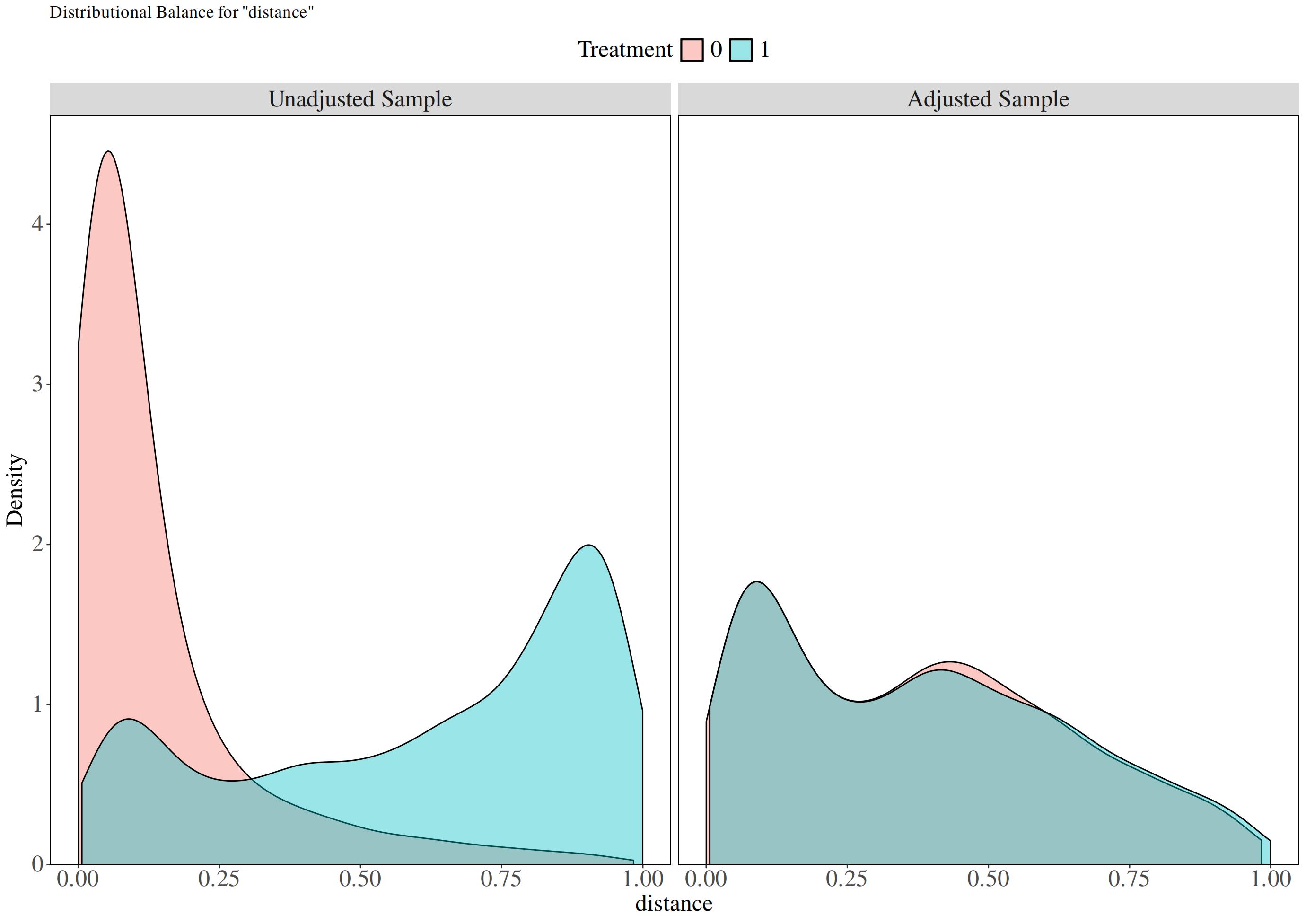

Supplement: Supplementary file 2 [file Image2.jpeg]

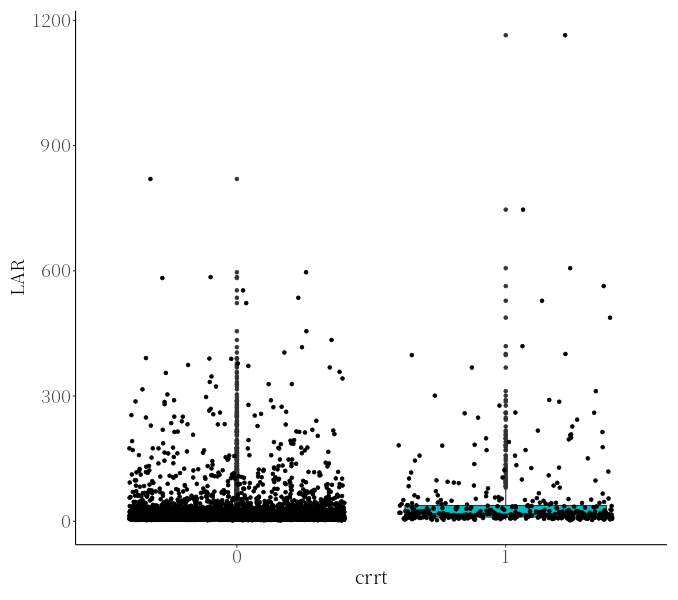

Supplement: Supplementary Figure/Image S3 — Showed the distribution of LAR across different subgroups (1 represents the crrt group and 0 represents the non-crrt group). [file Image3.png]
