# Supplementary material for: Association between lactate dehydrogenase to albumin ratio and ICU mortality in patients with acute kidney injury: a retrospective cohort study
Source: Front Nephrol. 2025 Jun 2;5:1583913. doi: 10.3389/fneph.2025.1583913 (PMC12171449; doi:10.3389/fneph.2025.1583913)
Supplement: SUPPLEMENTARY TABLE 1 — A multi-factor logistic regression analysis. [file Table1.docx]

Table S1

Analysis of the risk factors for hospital mortality in patients with AKI (univariate and multivariate logistic regression analyses)

| Variables | Univariate analysis | | | | |  | Multivariate analysis | | | | |
| --- | --- | --- | --- | --- | --- | --- | --- | --- | --- | --- | --- |
|  | β | S.E | Z | *P* | OR (95%CI) |  | β | S.E | Z | *P* | OR (95%CI) |
| Gender |  |  |  |  |  |  |  |  |  |  |  |
| F |  |  |  |  | 1.00 (Reference) |  |  |  |  |  |  |
| M | 0.01 | 0.06 | 0.16 | 0.870 | 1.01 (0.90 ~ 1.13) |  |  |  |  |  |  |
| Age | 0.01 | 0.00 | 6.87 | <.001 | 1.01 (1.01 ~ 1.02) |  | 0.01 | 0.00 | 4.14 | <.001 | 1.01 (1.01 ~ 1.02) |
| AKI stage |  |  |  |  |  |  |  |  |  |  |  |
| 1 |  |  |  |  | 1.00 (Reference) |  |  |  |  |  | 1.00 (Reference) |
| 2 | 0.14 | 0.07 | 2.05 | 0.040 | 1.15 (1.01 ~ 1.31) |  | 0.16 | 0.08 | 2.08 | 0.037 | 1.17 (1.01 ~ 1.36) |
| 3 | 1.43 | 0.07 | 19.20 | <.001 | 4.16 (3.60 ~ 4.81) |  | 1.04 | 0.09 | 11.26 | <.001 | 2.82 (2.35 ~ 3.37) |
| SOFA | 0.14 | 0.01 | 19.49 | <.001 | 1.15 (1.13 ~ 1.16) |  |  |  |  |  |  |
| APSIII | 0.03 | 0.00 | 23.45 | <.001 | 1.03 (1.03 ~ 1.03) |  | 0.02 | 0.00 | 13.91 | <.001 | 1.02 (1.02 ~ 1.03) |
| Charlson Comorbidity Index | 0.11 | 0.01 | 12.22 | <.001 | 1.12 (1.10 ~ 1.14) |  | 0.04 | 0.02 | 2.22 | 0.027 | 1.04 (1.01 ~ 1.08) |
| Myocardial Infarct | 0.03 | 0.07 | 0.42 | 0.671 | 1.03 (0.90 ~ 1.18) |  |  |  |  |  |  |
| Cerebrovascular Disease | 0.18 | 0.07 | 2.50 | 0.013 | 1.20 (1.04 ~ 1.38) |  | 0.43 | 0.09 | 4.68 | <.001 | 1.53 (1.28 ~ 1.83) |
| Chronic Pulmonary Disease | 0.06 | 0.06 | 0.96 | 0.339 | 1.06 (0.94 ~ 1.20) |  |  |  |  |  |  |
| Diabetes with Cc | -0.01 | 0.09 | -0.09 | 0.925 | 0.99 (0.83 ~ 1.18) |  |  |  |  |  |  |
| Congestive Heart Failure | 0.02 | 0.06 | 0.30 | 0.768 | 1.02 (0.91 ~ 1.14) |  |  |  |  |  |  |
| Severe Liver Disease | 0.64 | 0.08 | 8.36 | <.001 | 1.89 (1.63 ~ 2.19) |  | 0.42 | 0.11 | 3.81 | <.001 | 1.52 (1.22 ~ 1.88) |
| Metastatic Solid Tumor | 0.74 | 0.10 | 7.75 | <.001 | 2.11 (1.74 ~ 2.54) |  | 0.47 | 0.15 | 3.16 | 0.002 | 1.59 (1.19 ~ 2.13) |
| CKD | 0.60 | 0.09 | 6.43 | <.001 | 1.82 (1.51 ~ 2.18) |  |  |  |  |  |  |
| Sepsis | 1.02 | 0.08 | 12.37 | <.001 | 2.78 (2.36 ~ 3.27) |  |  |  |  |  |  |
| Antibiotic use | 1.92 | 0.06 | 30.78 | <.001 | 6.84 (6.05 ~ 7.73) |  | 1.87 | 0.07 | 27.06 | <.001 | 6.46 (5.64 ~ 7.39) |
| Vasopressor use | 0.84 | 0.06 | 14.43 | <.001 | 2.32 (2.07 ~ 2.60) |  | 0.43 | 0.07 | 6.08 | <.001 | 1.53 (1.33 ~ 1.76) |
| CRRT use | 1.09 | 0.10 | 10.56 | <.001 | 2.97 (2.43 ~ 3.64) |  | 0.26 | 0.13 | 1.99 | 0.046 | 1.30 (1.01 ~ 1.69) |
| LAR | 0.01 | 0.00 | 6.85 | <.001 | 1.01 (1.01 ~ 1.01) |  | 0.00 | 0.00 | 1.88 | 0.045 | 1.00 (1.00 ~ 1.00) |
| Wbc | 0.01 | 0.00 | 4.86 | <.001 | 1.01 (1.01 ~ 1.02) |  | 0.01 | 0.00 | 2.25 | 0.025 | 1.01 (1.01 ~ 1.01) |
| Hb | -0.10 | 0.01 | -8.02 | <.001 | 0.90 (0.88 ~ 0.93) |  | -0.04 | 0.02 | -2.50 | 0.012 | 0.96 (0.93 ~ 0.99) |
| Plt | -0.01 | 0.00 | -6.13 | <.001 | 0.99 (0.99 ~ 0.99) |  | -0.01 | 0.00 | -2.83 | 0.005 | 0.99 (0.99 ~ 0.99) |
| Bun | 0.01 | 0.00 | 10.89 | <.001 | 1.01 (1.01 ~ 1.01) |  | 0.01 | 0.00 | 4.59 | <.001 | 1.01 (1.01 ~ 1.01) |
| Scr | 0.06 | 0.02 | 4.04 | <.001 | 1.07 (1.03 ~ 1.10) |  | -0.25 | 0.03 | -7.37 | <.001 | 0.78 (0.73 ~ 0.83) |
| Glucose | -0.00 | 0.00 | -0.66 | 0.511 | 1.00 (1.00 ~ 1.00) |  |  |  |  |  |  |
| Potassium | 0.14 | 0.03 | 4.89 | <.001 | 1.15 (1.08 ~ 1.21) |  |  |  |  |  |  |
| Urineoutput | -0.01 | 0.00 | -11.44 | <.001 | 0.99 (0.99 ~ 0.99) |  |  |  |  |  |  |
